# Efficacy of mesenchymal stem cells in the treatment of peritoneal fibrosis in animal models: a systematic review and meta-analysis

**DOI:** 10.1080/0886022X.2024.2438863

**Published:** 2024-12-16

**Authors:** Lingqian Zheng, Wenmin Chen, Kaijin Yao, Yina Xie, Chunling Liao, Yongda Lin, Tianbiao Zhou

**Affiliations:** Department of Nephrology, the Second Affiliated Hospital of Shantou University Medical College, Shantou, China

**Keywords:** Peritoneal fibrosis, mesenchymal stem cells, animal models, meta-analysis

## Abstract

**Background:**

Peritoneal fibrosis is a serious complication of long-term peritoneal dialysis, often resulting in functional deterioration and withdrawal from therapy. Mesenchymal stem cells (MSCs) have demonstrated immunomodulatory and antifibrotic effects in various models. This meta-analysis evaluated the efficacy of MSCs therapy in animal models of peritoneal fibrosis.

**Methods:**

A comprehensive search of PubMed, the Cochrane Library, Web of Science, and EMBASE was conducted for studies published up to April 27, 2024. Two independent reviewers (LQZ and WMC) screened studies for inclusion, extracted data, and analyzed outcomes using RevMan 5.3 and STATA 17.0.

**Result:**

Fifteen studies met the inclusion criteria. MSC therapy significantly reduced inflammatory cytokines, including IL-6, TGF-β (SMD = −1.79, 95% CI: −2.32, −1.25, *p* < 0.00001), and TNF-α (SMD = −1.57, 95% CI: −2.71, −0.44, *p* = 0.006) levels. Additionally, MSCs reduced submesothelial thickness (MD = −63.14, 95% CI: −78.52, −47.76, *p* < 0.00001), Collagen I and Collagen III levels. MSCs treatment also improved ultrafiltration capacity (MD = 1.21, 95% CI: 0.64, 1.77, *p* < 0.0001), D/D0 of glucose and E-cadherin levels. However, no significant differences were observed in VEGF, D/P of Na, D/P of BUN, D/P of protein, or glucose mass transfer between the MSCs treatment group and the control group.

**Conclusion:**

MSC therapy significantly improves peritoneal function and attenuates fibrotic and inflammatory responses in animal models. These findings highlight the potential of MSCs as a promising therapeutic strategy for managing peritoneal fibrosis in clinical settings.

## 1. Introduction

Peritoneal dialysis (PD) is an important therapy option for people with end-stage renal failure [1]. However, long-term PD can lead to peritoneal fibrosis (PF) and ultrafiltration failure. More than 196,000 individuals with end-stage renal disease worldwide are presently undergoing PD treatment [1]. PD confers several benefits over traditional hemodialysis, including simple applications, efficient elimination of mesomolecular compounds, little impairment of residual renal function, and consistent hemodynamics, all of which contribute to preserving medical resources and associated costs [2]. Nevertheless, a high glucose concentration in the dialysis solution would result in changes in the morphology of the peritoneal tissue and deterioration of the peritoneal function, thus forcing patients to withdraw from PD [3]. PF is a complication of long-term PD, and its pathophysiological changes include an increase in α-SMA (α-Smooth muscle actin), collagen deposition, and thickening of the submesothelial thickness [4,5]. Researchers suggest controlling PF could improve peritoneal function and enhance survival in end-stage renal disease patients. Currently, slowing down PF involves using low GDPs and neutral pH, sparing glucose and metabolically active osmolytes, adding pharmacological agents to conventional PD solutions, and targeting glycolytic and pyruvate metabolism [6]. Nevertheless, there is currently a scarcity of safe and efficient treatment for PF patients.

Mesenchymal stem cells (MSCs) are a type of multipotent adult stem cell that can be obtained from various tissues, such as bone marrow, adipose tissue, umbilical cord blood, and placenta [7–9]. Many studies have proved that MSCs have regenerative, immunomodulatory, and antifibrotic capabilities. Various animal experiments investigated that MSCs can alleviate lung, heart, liver, and kidney fibrosis [10–12]. Researchers have recently found that MSCs can reduce PF and improve peritoneal function in PF animal models and patients. Due to the very small number of clinical studies on MSC treatment of PF, it cannot conduct a meta analysis of the PF patients, so we conducted a meta-analysis in animal models to evaluate the effectiveness of the MSCs, which could potentially provide crucial insights for future clinical trials.

Previously, we reviewed the clinical and preclinical studies of MSCs to alleviate PF and found that most of the studies showed MSCs improving peritoneal function and reducing PF, thus improving the life quality of PD patients [13]. In this meta-analysis, we collected data from the animal experiments and use the analysis software to further analyze the correlation between MSCs and PF. We have summarized the indicators of peritoneal function, such as ultrafiltration; D/P of Na (dialysate-to-plasma ratio of sodium); D/P of BUN (dialysate-to-plasma ratio of blood urea nitrogen); D/P of Cr (dialysate-to-plasma ratio of creatinine concentration); D/P of protein (dialysate-to-plasma ratio of protein); D/D0 of glucose (the Peritoneal absorption of glucose from the dialysate); and glucose mass transfer. Additionally, mesenchymal and fibrotic markers, such as α-SMA, Collagen I, Collagen III, submesothelial thickness, E-cadherin, fibronectin, and Snail, as well as cytokines and chemokines, including VEGF (vascular endothelial growth factor), IL-1β (interleukin-1β), IL-6 (interleukin-6), and TNF-α (tumor necrosis factor-alpha), were evaluated to assess the efficacy of MSCs in treating animal models of PF.

## 2. Materials and methods

### 2.1. Search strategy

**Figure 1.**
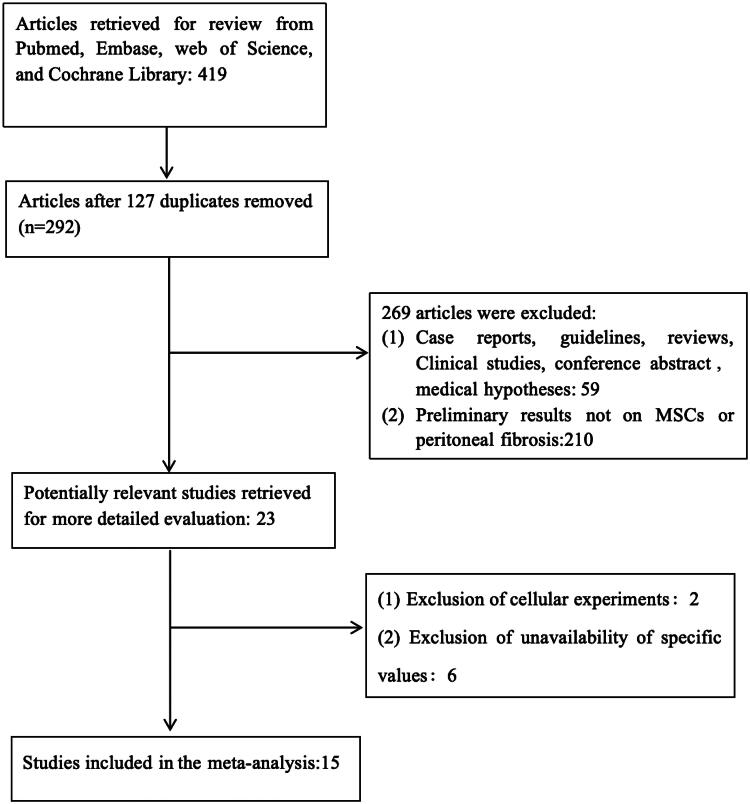
This flow chart illustrates the process of selecting literature for this meta-analysis.

The Cochrane Library, Embase, PubMed, and Web of Science databases were searched extensively from the inception to April 27, 2024, using the following search terms: (mesenchymal stromal cells OR mesenchymal stem cells OR mesenchymal progenitor cell OR multipotent stromal cells OR multipotent progenitor cell OR MSCs OR stem cells) AND (peritoneal fibrosis OR peritoneal sclerosis OR encapsulating peritoneal sclerosis OR peritoneal dialysis OR sclerosing peritonitis). The literature search was conducted by LQZ. We also critically reviewed the references of the included articles to confirm the inclusion of the full literature (Figure 1).

### 2.2. Inclusion and exclusion criteria

The following were the inclusion criteria: (1) the type of experiment was a randomized controlled animal experiment; (2) the subjects were mice or rats; (3) the experimental group represents PF animal models treated with MSCs, and the control group represents PF animal models treated with placebo or saline; (4) outcome: the efficacy of MSC in treating the PF models, which is measured by peritoneal function indicators, inflammation factors, and mesenchymal and fibrotic markers; (5) the literature included used English as the language;

The following were the exclusion criteria: (1) case reports, guidelines, reviews, clinical studies, conference abstracts, and medical hypotheses; (2) preliminary results not on MSCs or PF; (3) cellular experiments; and (4) Lack of specific values in the article.

### 2.3. Outcome measures

The recruited studies provided the following data on the efficiency of MSC treatment: D/P of Na, UF, D/P of BUN, D/P of Cr, D/P of protein, D/D0 of glucose, glucose mass transfer, fibronectin, TGF-β, α-SMA, E-cadherin, submesothelial thickness, Snail, Collagen III, Collagen I, IL-1β, IL-6, TNF-α, VEGF. If there were any disagreements, they were discussed and resolved with a third reviewer.

### 2.4. Quality assessment

Two independent reviewers (WMC and KJY) assessed each included study’s methodological quality using the SYRCLE Animal Experiment Bias Risk Assessment. Including ten components: (1) sequence generation; (2) Baseline characteristics; (3) Allocation concealment; (4) Random housing; (5) Researchers blinding; (6) Random outcome assessment; (7) Outcome assessors blinding; (8) Complete outcome data; (9) Outcome reporting; and (10) Other source of bias. The result of the assessment used ‘Yes’, ‘NO’, and ‘Unclear’ to represent a low-risk, high-risk, and unclear risk, respectively. The quality ratings of all the studies were summarized in Table 1, and the results were imported into Reveman software to produce a risk of bias map (Figure 3).

**Table 1.** Results of the risk of bias assessment of the included studies (SYRCLE animal experiment bias risk assessment).

Author, year	Sequence generation	Baseline characteristics	Allocation concealment	Random housing	Researchers blinding	Random outcome assessment	Outcome assessors blinding	Complete outcome data	Outcome reporting	Other sources of bias
Tülpar et al. 2012 [14]	Unclear	Yes	Unclear	Unclear	No	Unclear	Unclear	Yes	Yes	Yes
Baştuğ et al. 2013(1) [15]	Yes	Yes	Yes	Unclear	No	Unclear	Yes	Yes	Yes	Yes
Ueno et al. 2013 [16]	Unclear	Yes	Unclear	Unclear	No	Unclear	Yes	Yes	Yes	Yes
Baştuğ et al. 2013(2) [17]	Yes	No	Yes	Unclear	No	Unclear	Yes	Yes	Yes	Yes
KIM et al. 2014 [18]	Unclear	Yes	Unclear	Unclear	No	Yes	Unclear	Yes	Yes	Yes
Li et al. 2018 [19]	Yes	Yes	Unclear	Unclear	No	Unclear	Unclear	Yes	Yes	Yes
Zhou et al. 2019 [20]	Yes	Yes	Yes	Unclear	No	Unclear	Unclear	Yes	Yes	Yes
Costalonga et al. 2020 [21]	Unclear	Yes	Unclear	Unclear	No	Unclear	Unclear	Yes	Yes	Yes
Guo et al. 2020 [22]	Unclear	Yes	Unclear	Unclear	No	Unclear	Unclear	Yes	Yes	Yes
Setyo et al. 2020 [23]	Yes	Yes	Unclear	Unclear	No	Unclear	Unclear	Yes	Yes	Yes
Nagasaki et al. 2021 [24]	Unclear	Yes	Unclear	Unclear	No	Yes	Unclear	Yes	Yes	Yes
Yang et al. 2021 [25]	Unclear	Yes	Unclear	Unclear	No	Unclear	Unclear	Yes	Yes	Yes
Shi et al. 2022 [26]	Yes	Yes	Yes	Unclear	No	Unclear	Yes	Yes	Yes	Yes
Yu et al. 2023 [27]	Yes	Yes	Yes	Unclear	No	Yes	Unclear	Yes	Yes	Yes
Zhao et al. 2024 [28]	Yes	Yes	Unclear	Unclear	No	Unclear	Unclear	Yes	Yes	Yes

### 2.5. Statistical analysis

We gathered all relevant data on MSCs treating PF and analyzed the outcome using Review Manager Version 5.3 and STATA 17.0. I^2^ statistics and the Q test quantified heterogeneity due to study variation. The fixed effects model was used if the P value of the Q test was ≥ 0.1 or I^2^< 50%. Otherwise, the random effects model was applied to pool the outcomes. We used Weighted Mean Differences (WMD) or Standardized Mean Differences (SMD) to evaluate our collected continuous variables. The effect size of WMD was calculated if the outcome indicators’ units were the same, and the effect size of SMD was calculated if they were different. Moreover, the recruited investigations’ 95% confidence intervals (95% CI) were evaluated. We conducted sensitivity analyses to assess the stability of the included literature, subgroup analyses, and meta-regression to analyze the causes of the heterogeneity of the literature; we used funnel plots, Begg’s rank correlation test, and Egger’s linear regression test to identify publication bias.

## 3. Results

### 3.1. Search results

We searched the database and found 15 studies [14–28] in rats or mice related to MSCs treating PF. Figure 1 displays the search process flowchart. Table 2 presents the characteristics of the literature that was recruited.

**Table 2.** Characteristics of the studies included in this meta-analysis.

Author, year	n	Type of animal	Animal model	MSC type	Number of MSC	Route of delivery	Endpoints for this meta-analysis
Tülpar et al. 2012 [14]	*T* = 9, *C* = 9	Wistar albino rats	Peritoneal dialysis rat model	Rat-BM-MSCs	1.5 × 10^6^	IP	IL-6, TNF-ɑ, VEGF, TGF-β Submesothelial Thickness
Baştuğ et al. 2013 [15]	*T* = 9, *C* = 9	Wistar albino rats	HG-induced ultrafiltration failure in chronic peritoneal dialysis rat model	Rat-BM-MSCs	1.5 × 10^6^	IP	VEGF, D/P_Na_, UF, D/P _BUN_, D/P_Cr_, D/P_protein_, D/D0_glucose_, Glucose mass transfer, Submesothelial Thickness TGF-β
Ueno et al. 2013 [16]	*T* = 10, *C* = 10	Fisher 344 rats	CG-induced peritoneal fibrosis model	Rat-BM-MSCs	1 × 10^7^	IP	D/P _BUN_, D/D0_glucose_, ɑ-SMA,
Baştuğ et al. 2013 [17]	a: *T* = 9, *C* = 9 b: *T* = 8, *C* = 9	Wistar albino rats	Chronic peritoneal dialysis rat model	Rat-BM-MSCs	1.5 × 10^6^	a: IP b: IV	IL-6, TNF-ɑ, VEGF, D/P_Na_, UF, D/P _BUN_, D/P_Cr_, D/P_protein_, D/D0_glucose_, Glucose mass transfer, TGF-β, Submesothelial Thickness
KIM et al. 2014 [18]	*T* = 9, *C* = 9	Sprague-Dawley rats	Zy/scraping peritonitis	Rat-ADSCs	6 × 10^6^	IP	Submesothelial Thickness
Li et al. 2018 [19]	*T* = 12, *C* = 12	Wistar rats	MGO-induced peritoneal fibrosis model.	hUMSCs	2 × 10^6^	IV	UF, D/P_Cr_, ɑ-SMA, D/D0_glucose_, E-cadherin, Snail 1, TGF-β, Submesothelial Thickness,
Zhou et al. 2019 [20]	*T* = 9, *C* = 9	Wistar albino rats	Chronic peritoneal dialysis rat model	human-pMSCs	1.2–1.5 × 10^6^	IP	UF, Submesothelial Thickness
Costalonga et al. 2020 [21]	*T* = 8, *C* = 8	Wistar rats	Peritoneal fibrosis model developed in uremic rats	Rat-ADSCs	1 × 10^6^	IV	IL-6, TNF-ɑ, Fibronectin, TGF-β, CollagenIII, ɑ-SMA, IL-1β Submesothelial Thickness
Guo et al. 2020 [22]	*T* = 8, *C* = 8	Wistar rats	MGO-PD-induced rat peritoneal fibrosis model	hUMSCs	1 × 10^7^	IV	IL-6, TNF-ɑ, UF, D/P_Cr_, D/D0_glucose_, Fibronectin, TGF-β , IL-1β, ɑ-SMA, Snail1, Collagen III, Submesothelial Thickness
Setyo et al. 2020 [23]	*T* = 6, *C* = 6	Wistar albino rats	Peritoneal adhesion rat model	Rat-UCMSCs	a: 3 × 10^6^ b: 1.5 × 10^6^	Submucosal injection	TGF-β
Nagasaki et al. 2021 [24]	*T* = 5, *C* = 5	Sprague-Dawley (SD) rats	CG-induced peritoneal fibrosis model	human-BMSCs	5 × 10^6^	IP	D/P _BUN_, D/D0_glucose_, Submesothelial Thickness, ɑ-SMA, TGF-β, CollagenIII, CollagenI
Yang et al. 2021 [25]	*T* = 6, *C* = 6	Sprague-Dawley (SD) rats	Peritoneal fibrosis rat model	human-ADSCs human-BMSCs	1.5 × 10^6^	IP	Submesothelial Thickness
Shi et al. 2022 [26]	*T* = 6, *C* = 6	Sprague-Dawley (SD) rats	Peritoneal adhesion rat model	Rat-ADSCs-EV	400ug	IV	IL-6, Fibronectin, ɑ-SMA, E-cadherin
Yu et al. 2023 [27]	*T* = 6, *C* = 6	C57BL/6 mice	Peritoneal injury model	Rat-BM-MSCs-Exo	200 ug/kg	IP	IL-6, TNF-ɑ, VEGF, UF, D/P _BUN_, D/D0_glucose_, IL-1β, Fibronectin, Submesothelial Thickness, ɑ-SMA, TGF-β, CollagenIII,
Zhao et al. 2024 [28]	*T* = 6, *C* = 6	C57BL/6J mice	peritoneal fibrosis murine model	Rat-BM-MSCs-Exo	200 µg/kg	IP	UF, D/P_BUN_, D/D0_glucose,_ Fibronectin, Submesothelial Thickness, ɑ-SMA, E-cadherin, Snail, TGF-β, Collagen I

*Abbreviation:* Baştuğ et al. 2013 (1) and Baştuğ et al. 2013 (2): There are two papers of the author, so we used (1) and (2) to indicate it; T: MSC treatment group, which represents peritoneal fibrosis animal models treated with mesenchymal stem cells; C: control group, which stands for peritoneal fibrosis animal models treated with placebo or saline; hUMSCs: human umbilical cord blood mesenchymal stem cells; Rat-BM-MSC: rat bone marrow mesenchymal stem cells; ADSC: adipose-derived mesenchymal stem cells; Exo: exosome.

### 3.2. Characteristics of included literature

**Figure 2.**
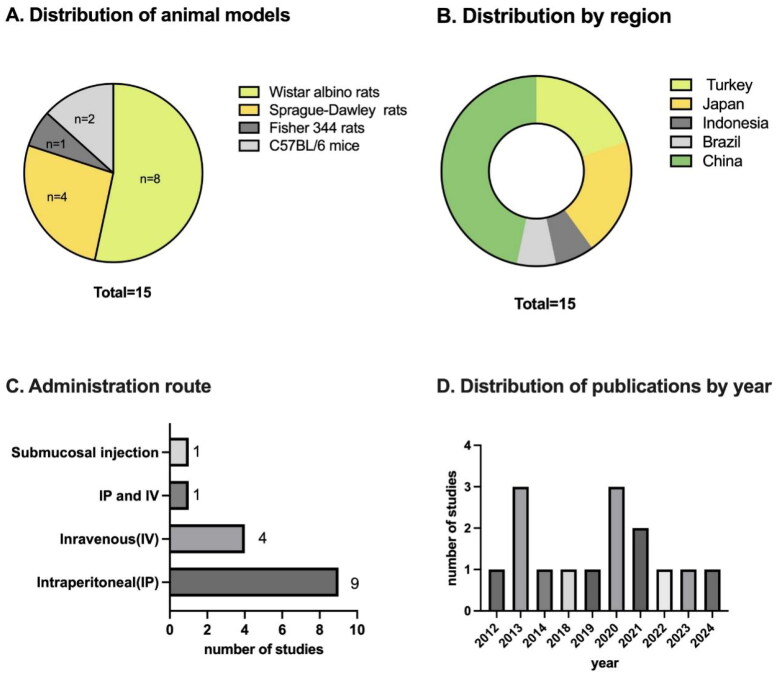
This figure covers the types of experimental animals, the distribution of countries and years, and the administration route of the MSCs in the included literature.

The experimental animal models included in the meta-analysis were different, eight studies used Wistar albino rats, four studies used Sprague-Dawley (SD) rats, two studies used C57BL/6 mice, and one study used Fisher 344 rats as the experimental animals. The injection methods in the included literature were different, nine studies used intraperitoneal injection (IP), four studies used intravenous injection (IV), one study used IP and IV injection, and one study used submucosal injection. In addition, the source of the MSC is varied, five studies used Rat-BM-MSC, and two studies used Rat-BMSC-Exo; four studies used Rat-ADSC, and one study used Rat-ADSC-EV; two studies used hUMSC, one study used human-pMSC and one study used Rat-UCMSC. Different studies included various methods to induce animal models, such as high glucose in the PD solution-induced PF rat models, CG-induced PF models, and MGO-induced PF rat models. Different studies use different injection dosages; this meta-analysis includes four different dosages: dose 1, which means injecting 1–2 × 10^6^ MSC; dose 2, which means injecting 5–6 × 10^6^ MSC; dose 3, which means injecting 1 × 10^7^ MSC; and dose 4, which means injecting 200 µg/kg MSC (Figure 2).

### 3.3. Quality assessment

**Figure 3.**
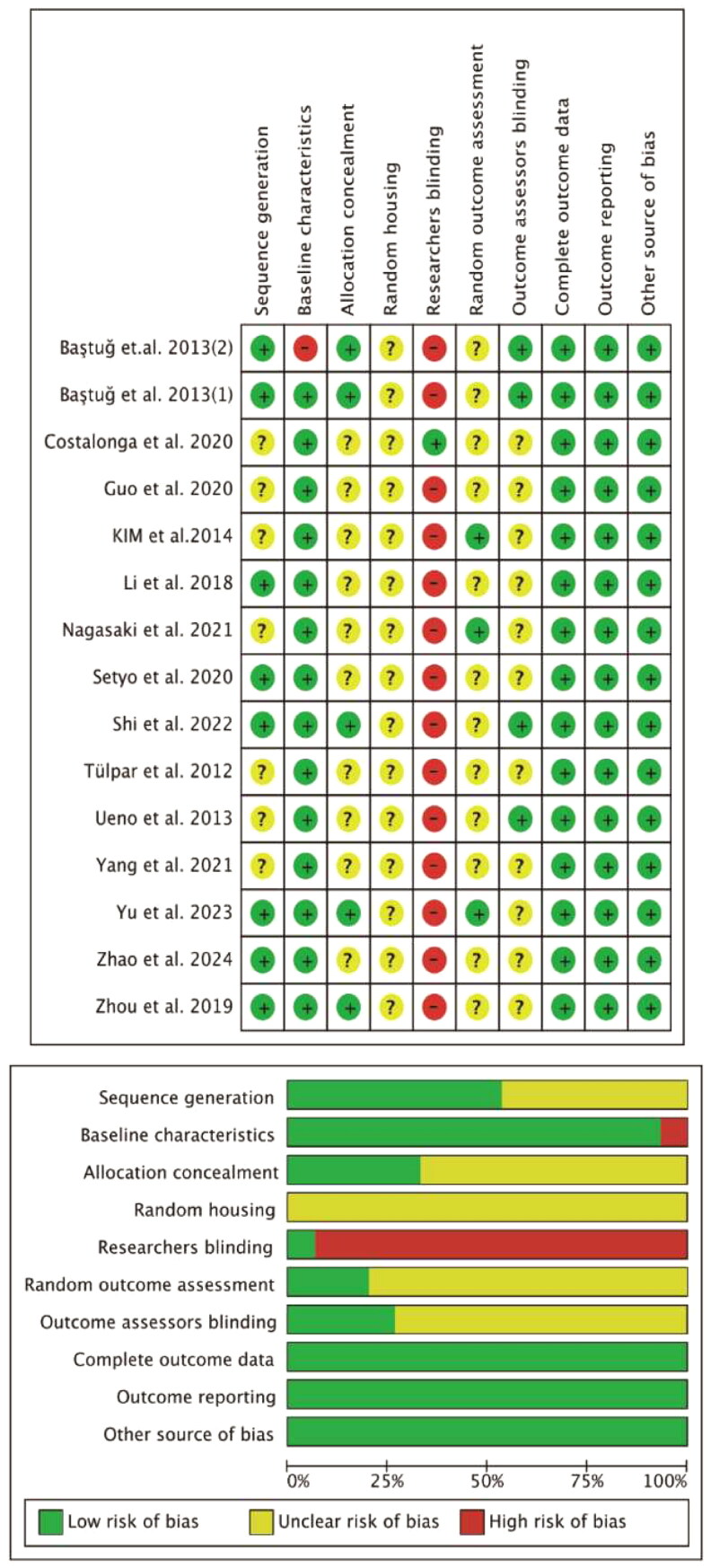
Aggregate risk of bias graph for each experimental animal study and risk of bias summary.

We used the SYRCLE Animal Experiment Bias Risk Assessment to assess the methodological quality of recruited studies. The majority of the included studies were rated as having a low or unclear risk of bias, but the risk of researchers blinding is high, which can be explained by the fact that most of the included studies did not blind the researchers and animal keepers. Figure 3 summarizes the risk of bias map in the included studies.

### 3.4. Assessment of cytokines

Long-term exposure to the PD fluid would affect the peritoneal function and structure [29], because the high concentrations of glucose in the dialysis fluid would stimulate mesothelial cells and macrophages to release a series of inflammatory cytokines such as IL-6, IL-8, TNF-α and growth factor such as VEGF and TGF-β [30]. A retrospective cohort has shown that PD-related peritonitis has a significant correlation to mortality in PD patients when they are dialyzed for more than two years [31]. So it is important for us to analyze the changes of cytokines when treating with MSCs.

#### 3.4.1. IL-6

**Figure 4.**
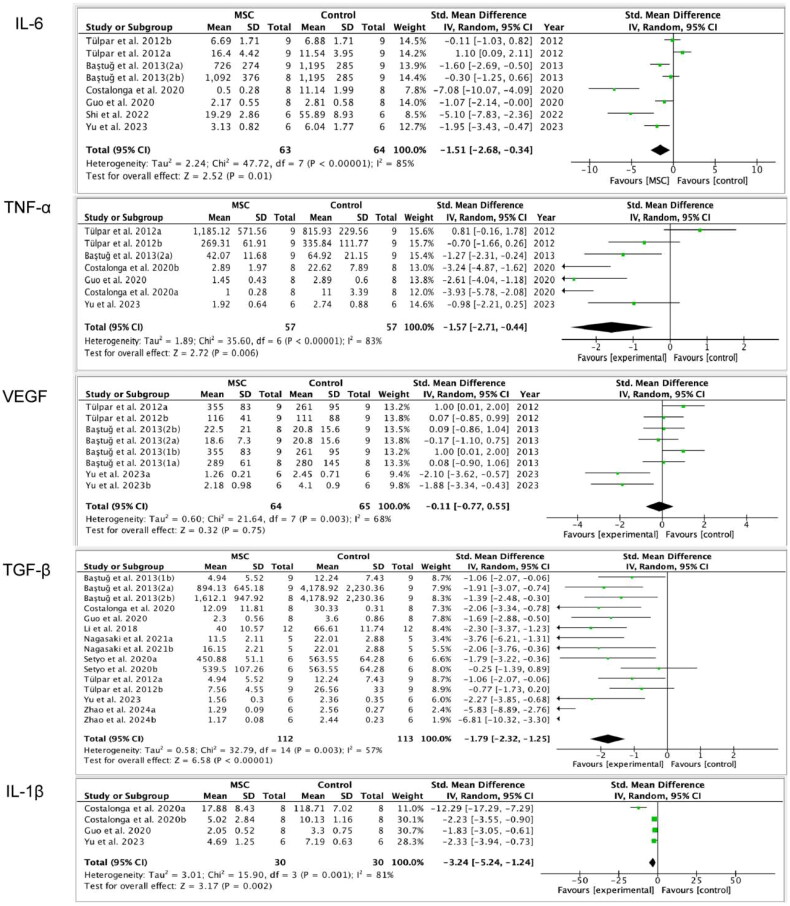
The efficacy of mesenchymal stem cells (MSCs) on IL-6 (interleukin 6), TNF-α (tumor necrosis Factor-alpha), VEGF (vascular endothelial-derived growth factor), TGF-β (transforming growth factor-β), IL-1β (interleukin-1β). This figure indicates that the MSCs therapy group reduces IL-6, TNF-α, TGF-β and IL-1β levels. However, there is no significant difference in VEGF levels between the MSCs treatment group and the control group.

Six studies [14,17,21,22,26,27] were conducted to assess the efficacy of MSC on IL-6, and the findings demonstrated that in the PF rat models, the MSC treatment group had lower levels of IL-6 compared to the control group (SMD= −1.51, 95%CI: −2.68, −0.34; *p* = 0.01; Table 3). In addition, we divided the data into two subgroups based on injection modality. The result shows that there was no statistical significance in IL-6 between the MSC treatment group and control group while using IP injection (SMD= −0.58, 95% CI: −1.92, 0.76; *p* = 0.40; Table 3). But, while using IV injection, the level of IL-6 in the MSC treatment group is lower than the control group (SMD= −2.94, 95%CI: −5.26, −0.61; *p* = 0.01; Table 3), which indicates that IV injection of MSCs may help to down-regulate IL-6 (Figure 4).

**Table 3.** Meta-analysis of the efficacy of MSC in the therapy of peritoneal fibrosis.

Indicators	Sub-group		Studies Number	Q test p value	Model Selected	SMD/MD (95%CI)	p
IL6	Overall		8	< 0.00001	Random	−1.51 [-2.68, −0.34]	0.01
	Injection method	IP	4	0.0005	Random	−0.58 [-1.92, 0.76]	0.40
		IV	4	< 0.00001	Random	−2.94 [-5.26, −0.61]	0.01
IL-1β	Overall		4	0.001	Random	−3.24[-5.24, −1.24]	0.02
TGF-β	Overall		15	0.003	Random	−1.79 [-2.32, −1.25]	< 0.00001
	Sources of MSCs	Rat-MSCs	11	0.003	Random	−1.66 [-2.31, −1.01]	< 0.00001
		Human-MSCs	4	0.51	Fixed	−2.17 [-2.86, −1.48]	< 0.00001
	Injection method	IP	9	0.002	Random	−2.14 [-3.02, −1.25]	< 0.00001
		IV	4	0.67	Fixed	−1.86 [-2.43, −1.28]	< 0.00001
	Experimental animal	Rat	12	0.16	Fixed	−1.44 [-1.78, −1.10]	< 0.00001
		mice	3	0.02	Random	−4.67 [-7.74, −1.59]	0.003
	Dose1	1–2 × 10^6^MSC	8	0.15	Fixed	−1.30 [-1.68, −0.92]	< 0.00001
	Dose4	200ug/kg/dose MSC	3	0.02	Random	−4.67 [-7.74, −1.59]	0.003
TNF-α	Overall		7	< 0.00001	Random	−1.57 [-2.71, −0.44]	0.006
	Injection method	IP	4	0.02	Random	−0.51 [-1.46, 0.43]	0.29
		IV	3	0.06	Random	−2.20 [-3.57, −0.84]	0.002
VEGF	Overall		8	0.003	Random	−0.11 [-0.77, 0.55]	0.75
D/P of Na	Overall		4	0.90	Fixed	0.01 [-0.02, 0.03]	0.62
UF	Overall		9	< 0.00001	Random	1.21 [0.64, 1.77]	< 0.0001
	Injection method	IP	6	< 0.00001	Random	1.14 [0.52, 1.76]	0.0003
		IV	3	0.96	Fixed	1.74 [0.44, 3.03]	0.009
	Sources of MSCs	Rat-MSCs	6	< 0.0001	Random	0.75 [0.20, 1.29]	0.007
		Human-MSCs	3	0.30	Fixed	2.41 [1.43, 3.38]	< 0.00001
	Experimental animal	Rats	7	0.01	Random	4.12 [1.92, 6.33]	0.0002
		Mice	2	0.04	Random	0.55 [0.32, 0.78]	< 0.00001
	Dose1	1–2 × 10^6^MSC	6	0.05	Random	5.28 [2.15, 8.41]	0.0009
	Dose4	200ug/kg/dose MSC	2	0.04	Random	0.55[0.32,0.78]	< 0.00001
D/P of BUN	Overall		9	< 0.00001	Random	−0.05 [-0.17, 0.06]	0.37
D/P of Cr	Overall		6	0.57	Fixed	−0.12 [-0.18, −0.06]	0.0002
	Injection method	IP	3	0.19	Fixed	−0.13 [-0.21, −0.05]	0.002
		IV	3	0.82	Fixed	−0.11 [-0.21, −0.01]	0.03
	Sources of MSCs	Rat-MSCs	4	0.29	Fixed	−0.12 [-0.19, −0.05]	0.001
		Human-MSCs	2	0.79	Fixed	−0.14 [-0.28, 0.00]	0.05
D/P of Protein	Overall		4	0.01	Random	−4.24 [-13.86, 5.39]	0.39
D/D0 of glucose	Overall		11	< 0.00001	Random	0.14 [0.05, 0.23]	0.004
	Injection method	IP	8	< 0.00001	Random	0.17 [0.05, 0.28]	0.005
		IV	3	0.44	Fixed	0.05 [-0.02, 0.12]	0.14
	Sources of MSCs	Rat-MSCs	7	< 0.00001	Random	0.22 [0.11, 0.33]	< 0.0001
		Human-MSCs	4	0.002	Random	−0.04 [-0.11, 0.04]	0.36
	Experimental animal	Rats	9	< 0.00001	Random	0.13 [0.03, 0.23]	0.01
		Mice	2	0.51	Fixed	0.15 [0.10, 0.21]	< 0.00001
	Dose1	1–2 × 10^6^MSC	5	< 0.00001	Random	0.32 [0.04,0.59]	0.02
	Dose3	1 × 10^7^MSC	2	0.71	Fixed	0..13 [0.06,0.20]	0.0007
	Dose4	200ug/kg/dose MSC	2	0.51	Fixed	0.15 [0.10,0.21]	<0.00001
Glucose mass transfer	Overall		4	0.0006	Random	−0.41 [-1.67, 0.85]	0.53
Fibronectin	Overall		8	< 0.00001	Random	−2.82 [-4.26, −1.38]	0.0001
	Injection method	IP	4	0.006	Random	−3.57 [-5.74, −1.40]	0.001
		IV	4	< 0.0001	Random	−2.14 [-4.14, −0.14]	0.04
Submesothelial thickness	Overall		16	< 0.00001	Random	−63.14 [-78.52, −47.76]	< 0.00001
	Injection method	IP	12	< 0.00001	Random	−59.61 [-77.35, −41.88]	< 0.00001
		IV	4	0.20	Fixed	−68.08 [-87.27, −48.90]	< 0.00001
	Sources of MSCs	Rat-MSCs	9	0.005	Random	−64.17 [-84.92, −43.42]	< 0.00001
		Human-MSCs	7	0.03	Random	−48.50 [-65.47, −31.54]	< 0.00001
	Experimental animal	Rats	14	< 0.00001	Random	−70.39 [-89.62, −51.16]	< 0.00001
		Mice	2	0.72	Fixed	−39.85 [-45.99, −33.71]	< 0.00001
	Dose1	1–2 × 10^6^MSC	10	< 0.00001	Random	−79.60 [-103.50,-55.70]	< 0.00001
	Dose2	5–6 × 10^6^MSC	3	0.08	Random	−44.74 [-96.61,7.12]	0.09
	Dose4	200ug/kg/dose MSC	2	0.717	Fixed	−39.85 [-45.99,-33.71]	< 0.00001
α-SMA	Overall		14	0.01	Random	−2.26 [-2.83, −1.68]	< 0.00001
	Injection method	IP	8	0.004	Random	−2.89 [-3.98, −1.80]	< 0.00001
		IV	6	0.40	Fixed	−1.88 [-2.38, −1.38]	< 0.00001
	Sources of MSCs	Rat-MSCs	8	0.009	Random	−2.65 [-3.61, −1.70]	< 0.00001
		Human-MSCs	6	0.21	Fixed	−1.87 [-2.40, −1.34]	< 0.00001
	Experimental animal	Rats	10	0.43	Fixed	−1.92 [-2.33, −1.51]	< 0.00001
		Mice	4	0.002	Random	−4.48 [-7.17, −1.78]	0.001
	Dose1	1–2 × 10^6^MSC	2	0.49	Fixed	−2.51 [-3.39,-1.64]	< 0.00001
	Dose3	1 × 10^7^MSC	5	0.71	Fixed	−1.60 [-2.10, −1.10]	< 0.00001
	Dose4	200ug/kg/dose MSC	4	0.002	Random	−4.48 [-7.17, −1.78]	0.001
E-cadherin	Overall		4	< 0.00001	Random	8.17 [1.32, 15.02]	0.02
Snail	Overall		5	< 0.00001	Random	−3.55 [-5.92, −1.19]	0.003
Collagen III	Overall		6	0.56	Fixed	−1.50[-2.05, −0.96]	< 0.00001
	Injection method	IP	4	0.004	Random	−6.96[-11.28, −2.65]	0.002
		IV	2	0.09	Random	−2.40[-6.38, 1.59]	0.24
Collagen I	Overall		4	0.07	Random	−2.51[-3.93,-1.09]	0.0005

*Abbreviations:* IP: intraperitoneal injection; IV: intravenous injection; IL-6: interleukin 6; VEGF: vascular endothelial-derived growth factor; D/P of Na: the dialysate to-plasma ratio of sodium; D/P of BUN: dialysate-to-plasma ratio of blood urea nitrogen; UF: ultrafiltration capacity; MSC: mesenchymal stem cell; D/P of Cr: the ratio of dialysate and plasma creatinine concentration; D/P of protein: the dialysate to-plasma ratio of protein; D/D0 of glucose: the peritoneal absorption of glucose from the dialysate; TGF-β: transforming growth factor-β; α-SMA: α-smooth muscle actin; IL-1β: interleukin-1β; Dose1: 1–2 × 10^6^MSC; Dose2: 5–6 × 10^6^MSC; Dose 3:1 × 10^7^MSC; Dose4: 200 µg/kg MSC.

#### 3.4.2. IL-1β

Three studies [21,22,27] were included to analyze IL-1β. The finding indicated that the MSC therapy group had a lower level of IL-1β compared to the control group (SMD = −3.24, 95%CI: −5.24, −1.24; *p* = 0.02; Table 3), which indicates that MSC treatment can down-regulate the level of IL-1β in PF animal models (Figure 4).

#### 3.4.3. TGF-β

Ten studies [14,15,17,19,21–24,27,28] were included to assess the level of TGF-β. The outcomes indicate that the MSC therapy group had lower levels of TGF-β than the control group (SMD= −1.79, 95%CI: −2.32, −1.25; *p* < 0.00001). Subgroup analysis was then carried out based on the MSC source, injection modality, experimental animals, and MSC dosages. As a result, IP injection and IV injection can lower the level of TGF-β. There is no difference in TGF-β levels when used in rats or mice as the experimental animals. Both Rat-MSC treatment and human-MSC treatment can decrease the TGF-β level. Injecting dose 1 or dose 4 MSC is no difference in down-regulating the level of TGF-β (Table 3; Figure 4).

#### 3.4.4. TNF-α

Five studies [14,17,21,22,27] were included to assess the level of TNF-α, and the outcomes demonstrate that the MSC treatment group’s TNF-α level was lower than the control group (SMD= −1.57, 95%CI: −2.71, −0.44; *p* = 0.006; Table 3). Then we performed a subgroup analysis according to the injection method, and we found that there is no statistical significance on TNF-α between the MSCs therapy group and control group when using IP injection (SMD= −0.51, 95%CI: −1.46, 0.43; *p* = 0.29; Table 3). However, the result is remarkable when IV injection is used (SMD = −2.20, 95%CI: −3.57, −0.84; *p* = 0.002; Table 3) (Figure 4).

#### 3.4.5. VEGF

Four studies [14,15,17,27] were included to detect the level of VEGF. The results reveal that there was no statistical difference in VEGF between the MSC treatment group and control group (SMD = −0.11, 95%CI: −0.77, 0.55; *p* = 0.75; Table 3) (Figure 4).

##### 3.4.6. Assessment of peritoneal function

Peritoneal membrane transport function was assessed by the dialysate-to-plasma ratio (D/P) of urea nitrogen, creatinine, sodium and protein, D/D0 of glucose, and Glucose mass transfer [15]. Peritoneal transport function has a substantial impact on PD efficiency in patients with fibrosis, as fibrosis affects ultrafiltration capacity and modifies capillary surface area, resulting in poorer fluid removal and decreased overall dialysis effectiveness [32].

#### 3.4.7. D/P of Na, D/P of BUN

**Figure 5.**
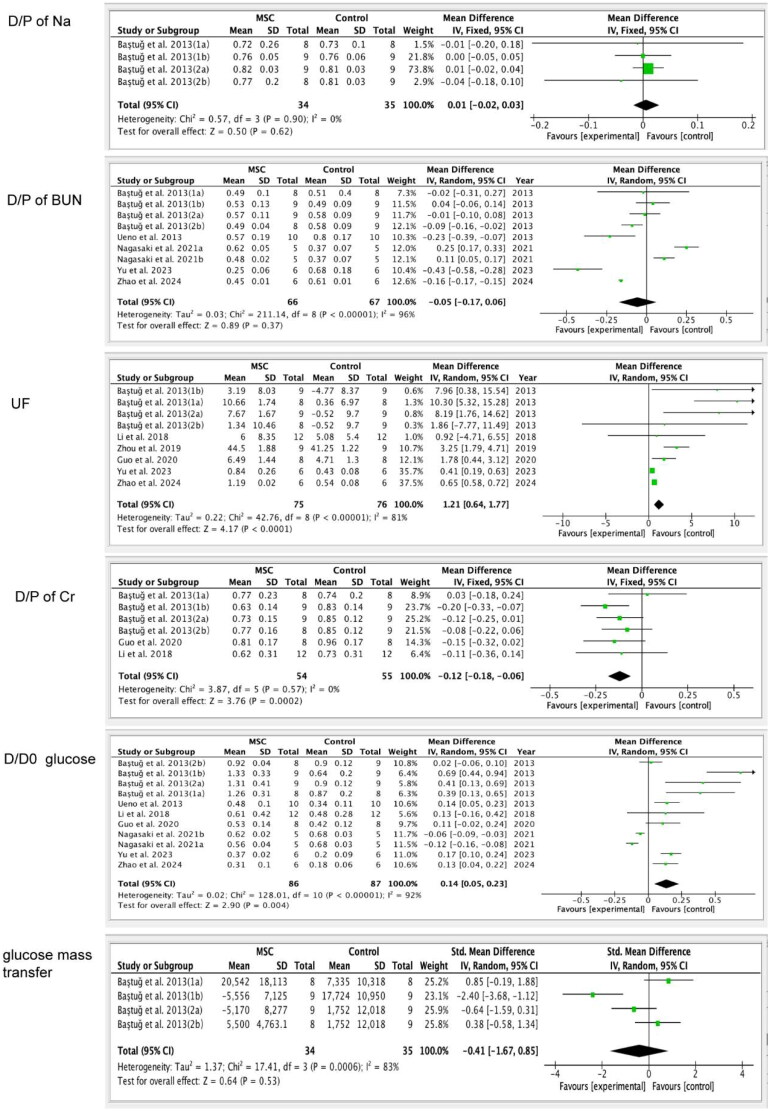
The efficacy of mesenchymal stem cells (MSCs) on D/P of Na (dialysate-to-plasma ratio of sodium), D/P of BUN (dialysate-to-plasma ratio of blood urea nitrogen), UF (ultrafiltration), D/P of Cr (dialysate-to-plasma ratio of creatinine), D/D0 of glucose (peritoneal absorption of glucose from the dialysate), and glucose mass transfer. This figure demonstrates that MSCs can improve UF levels and D/D0 of glucose in animal models of peritoneal fibrosis.

Two studies [15,17] were included to assess the peritoneal function of D/P of Na, and six studies [15–17,24,27,28] were incorporated to evaluate the peritoneal function of D/P of BUN. The results show that there were no discernible variations in D/P of Na (MD= 0.01, 95%CI: −0.02, 0.03; *p* = 0.62; Table 3) and D/P of BUN levels (MD= −0.05, 95%CI: −0.17, 0.06; *p* = 0.37; Table 3) between the MSC group and the control group (Figure 5).

#### 3.4.8. Ultrafiltration capacity (UF)

Seven studies [15,17,19,20,22,27,28] were included to assess the ultrafiltration capacity (UF) in PF rats and mice. According to the findings, the MSC treatment group exhibits a higher UF compared to the control group (MD = 1.21, 95% CI: 0.64, 1.77; *p* < 0.0001; Table 3), which indicates that MSC treatment may improve the UF in PF models. Then, we conducted the subgroup analysis according to injection methods, MSC dosages, sources of MSCs, and types of experimental animals. As a result, we do not find the difference between the IP injection (MD = 1.14, 95%CI: 0.52, 1.76; *p* = 0.0003) and IV injection (MD = 1.74, 95%CI: 0.44, 3.03; *p* = 0.009), and the two injection methods both can improve the UF in PF models. We also find no difference in UF between Rat-MSCs (MD = 0.75, 95%CI: 0.20, 1.29; *p* = 0.007; Table 3) and human-MSCs (MD = 2.41, 95%CI:1.43, 3.38; *p* < 0.00001; Table 3) in treating the PF rats/mice models. At the same time, there is no difference in UF between the types of experimental animals. Whether rats or mice are used as experimental animals, the UF in the MSC therapy group is higher than the control group (rats: MD = 4.12, 95%CI: 1.92, 6.33; *p* = 0.0002; mice: MD = 0.55, 95%CI: 0.32, 0.78; *p* < 0.00001). Injection of MSC at doses 1 and 4 resulted in a lower level of UF (Table 3; Figure 5).

#### 3.4.9. D/P of Cr

Four studies [15,17,19,22] were recruited to assess the D/P of Cr in PF rats and mice models. The result shows that the level of D/P of Cr in the MSC treatment group is lower than in the control group (MD= −0.12, 95%CI: −0.18, −0.06; *p* = 0.0002), and I^2^=0% indicate that low heterogeneity and reliable results. We also conducted subgroup analyses based on the injection method and the source grouping of MSCs. The results revealed that both IP injection (MD= −0.13, 95%CI: −0.21, −0.05; *p* = 0.002) and IV injection (MD= −0.11, 95%CI: −0.21, −0.01; *p* = 0.03) can reduce the level of D/P of Cr between the MSC treatment groups and control groups. In addition, there was no difference in the reduction of D/P of Cr between the Rat-MSCs treatment group (MD= −0.12, 95%CI: −0.19, −0.05; *p* = 0.001) and the human-MSCs treatment group (MD= −0.14, 95%CI: −0.28, 0.00; *p* = 0.05), both of which can reduce the D/P of Cr compared to the control group (Table 3; Figure 5).

#### 3.4.10. D/P of protein

Two studies [15,17] were included to assess the level of D/P of protein in PF models. The result shows no statistical significance in the level of D/P of protein in the MSC treatment group compared to the control group (MD= −4.24, 95%CI: −13.86, 5.39; *p* = 0.39; Table 3).

#### 3.4.11. D/D0 of glucose

Eight studies [15–17,19,22,24,27,28] were included to assess the level of D/D0 of glucose, and the result shows that the level of D/D0 of glucose in the MSC therapy group is higher than the control group (MD = 0.14, 95%CI: 0.05, 0.23; *p* = 0.004). Moreover, we made the subgroup analysis and found that IP injection can increase the level of D/D0 of glucose (MD = 0.17, 95%CI: 0.05, 0.28; *p* = 0.005), while IV injection (MD = 0.05, 95%CI: −0.02, 0.12; *p* = 0.14) was not notable between the MSC treatment group and the control group. We also made a subgroup analysis according to the sources of MSCs, and the result indicated that the level of D/D0 of glucose in the Rat-MSCs treatment (MD = 0.22, 95%CI:0.11, 0.33; *p* < 0.0001) is higher than the control group, while the difference between the human-MSCs treatment group (MD=-0.04, 95%CI: −0.11, 0.04; *p* = 0.36) and control group was not notable. At the same time, whether using rats or mice, we cannot find any differences in D/D0 of glucose between the MSC-treated group and the control group (rats: MD = 0.13, 95%CI: 0.03, 0.23; *p* = 0.01; mice: MD = 0.15, 95%CI: 0.10,0.21; *p* < 0.00001). Moreover, injection of MSC at doses 1, 3, and dose 4 resulted in decreased D/D0 of glucose levels (Table 3; Figure 5).

#### 3.4.12. Glucose mass transfer

Two studies [15,17] were included to assess the glucose mass transfer, and the findings indicate that there was no difference between the MSCs therapy group and the control group (MD= −0.41, 95%CI: −1.67, 0.85; *p* = 0.53; Table 3 and Figure 5).

##### 3.4.13. Assessment of mesenchymal and fibrotic markers

The process of PF consists of two parts, the fibrotic process and the inflammation, which are closely related and inseparable [33]. In this part, we will analyze the efficacy of mesenchymal and fibrotic markers (such as Fibronectin, α-SMA, E-cadherin and Snail) under MSCs treatment. Due to various biologically incompatible components in the PD fluid [4,34,35], peritoneal mesothelial cells would undergo a series of changes in morphological and histological when patients under long-term PD, such as the abnormal proliferation in the α-SMA, accumulation of Collagen I and Collagen III, suppress the expression of E-cadherin [2], and the thickness of submesothelial compact zone increases [4,5,29]. Therefore, it is significant for us to analyze the total effect of the mesenchymal and fibrotic markers when using MSCs in animal models.

#### 3.4.14. Fibronectin

**Figure 6.**
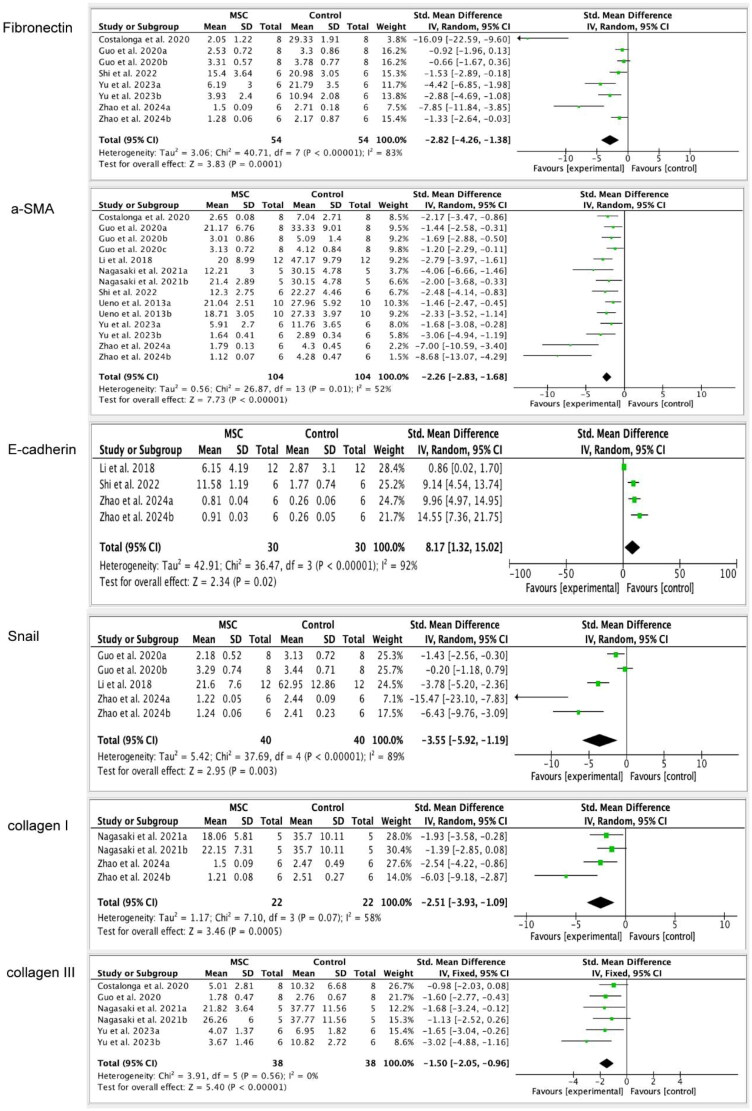
The efficacy of mesenchymal stem cells (MSCs) on Fibronectin, α-SMA (α-Smooth muscle actin), E-cadherin, Snail, Collagen I, Collagen III. The figure indicates that MSCs therapy can decrease the levels of α-SMA, Snail, Collagen I and Collagen III, while improving the levels of E-cadherin.

Five studies [21,22,26–28] were included to assess the level of Fibronectin. The result indicates that fibronectin levels in the MSC therapy group were found to be lower than those in the control group (SMD= −2.82, 95%CI: −4.26, −1.38; *p* = 0.0001). We made the subgroup analysis according to the injection method, and the outcome indicates both IP and IV injection can reduce the level of Fibronectin (IP injection: SMD= −3.57, 95%CI: −5.74, −1.40; *p* = 0.001; IV injection: SMD= −2.14, 95%CI: −4.14, −0.14; *p* = 0.04; Table 3 and Figure 6).

#### 3.4.15. Submesothelial thickness

**Figure 7.**
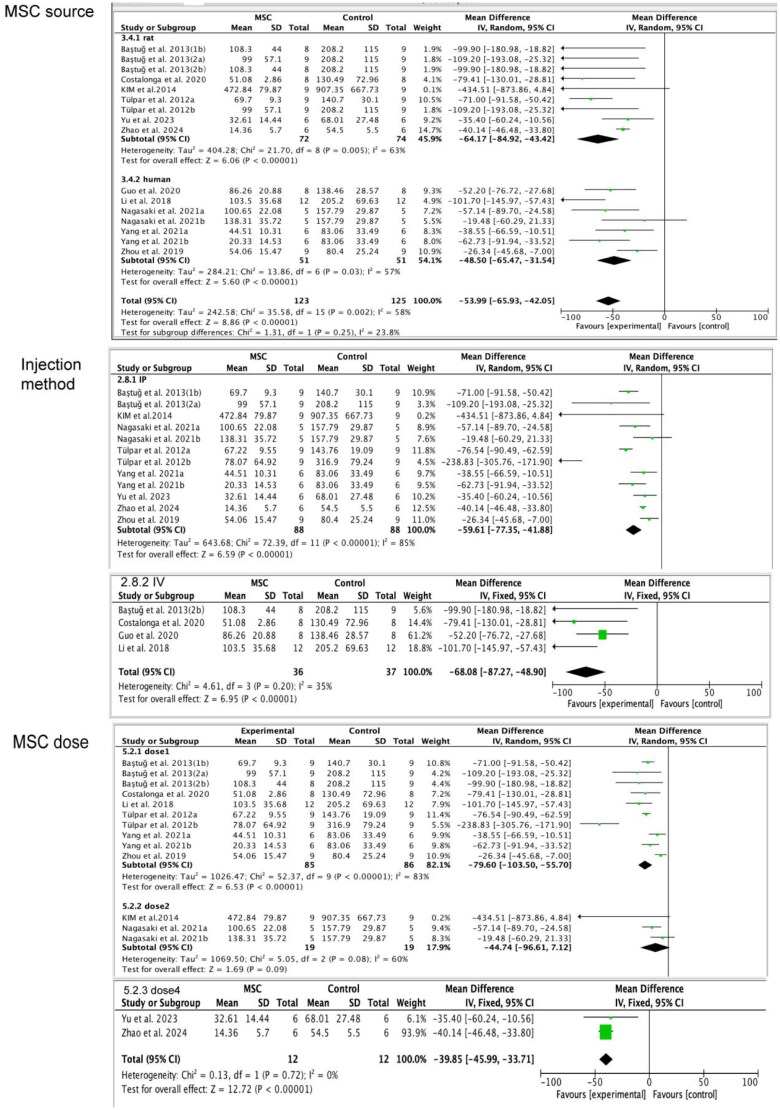
Subgroup analysis of the submesothelial thickness (SMC), part 1 of figure 7 indicates that rat-MSC and human-MSC both can decrease the submesothelial thickness; part 2 of figure 7 indicates that there is no difference between the IP injection and IV injection in decreasing the submesothelial thickness; part 3 of figure 7 indicates that dose 1 and dose 4 of MSC can decrease the submesothelial thickness, but there is no statistical significance in dose 2 between the MSC treatment group and control group.

Twelve studies [14,15,17–22,24,25,27,28] were included to assess the submesothelial thickness in the PF rats and mice models. The result shows that MSCs treatment groups had lower submesothelial thickness compared to the control group (MD= −63.14, 95%CI: −78.52, −47.76; *p* < 0.00001). Then, we conducted the subgroup analysis according to the injection methods, sources of MSCs, dosage of MSC, and experimental animals. As a result, we found IP injection (MD= −59.61, 95%CI: −77.35, −41.88; *p* < 0.00001) and IV injection (MD= −68.08, 95%CI: −87.27, −48.90; *p* < 0.00001) both can reduce the submesothelial thickness in the PF models. Both Rat-MSCs treatment (MD= −64.17, 95%CI: −84.92,-43.42; *p* < 0.00001) and human-MSCs treatment (MD= −48.50, 95%CI: −65.47, −31.54; *p* < 0.00001) can reduce the submesothelial thickness in the PF models, and whether used the rats or mice as the experimental animals; the submesothelial thickness can be decreased in the PF models (rats: MD= −70.39, 95%CI: −89.62, −51.16; *p* < 0.00001; mice: MD= −39.85, 95%CI: −45.99,-33.71; *p* < 0.00001). As for MSC dosage, dose 1 and dose 4 could reduce the submesothelial thickness, but dose 2 is not notable among the MSC therapy group and control group (dose 1: MD: −79.60, 95%CI: −103.50, −55.70; *p* < 0.00001; dose 2: MD: −44.74, 95%CI: −96.61,7.12; *p* = 0.09; dose 4: MD: −39.85, 95%CI: −45.99, −33.71; *p* < 0.00001; Table 3 and Figure 7).

#### 3.4.16. ɑ-SMA

Eight studies [16,19,21,22,24,26–28] were included to assess the level of α-SMA. The result shows that the α-SMA level in the MSC treatment group is lower than the control group (SMD= −2.26, 95%CI: −2.83, −1.68; *p* < 0.00001). After that, we conducted a subgroup analysis based on the injection methods, sources of MSCs, MSC dosages, and experimental animals. We found that there was no difference between the IP injection (SMD= −2.89, 95%CI: −3.98, −1.80; *p* < 0.00001) and IV injection (SMD= −1.88, 95%CI: −2.38, −1.38; *p* < 0.00001) to reduce the α-SMA in PF models. Both the Rat-MSC treatment group (SMD= −2.65, 95%CI: −3.61, −1.70; *p* < 0.00001) and human-MSC treatment group (SMD= −1.87, 95%CI: −2.40, −1.34; *p* < 0.00001) can reduce the α-SMA level compared to the control group. Whether rats or mice are used as experimental animals, the level of α-SMA is lower in the MSC-treated group than in the control group (rats: SMD= −1.92, 95%CI: −2.33, −1.51; *p* < 0.00001; mice: SMD= −4.48, 95%CI: −7.17, −1.78; *p* = 0.001). Moreover, injecting MSC at doses of 1,3 and 4 resulted in a decrease in α-SMA levels (Table 3; Figure 6).

#### 3.4.17. E-cadherin and Snail

Three studies [19,26,28] were included to evaluate the E-cadherin level. The result shows that the MSC treatment group can increase the E-cadherin level compared to the control group (SMD= 8.17, 95%CI: 1.32, 15.02; *p* = 0.02). Five studies were included to assess the Snail level. The outcome indicated that the Snail level in the MSC treatment group is lower than that in the control group (SMD= −3.55, 95%CI: −5.92, −1.19; *p* = 0.003; Table 3 and Figure 6).

#### 3.4.18. Collagen I, Collagen III

Two studies [16,28] were included to assess the level of Collagen I. The outcome demonstrates that, in comparison to the control group, the MSC therapy group can lower the amount of Collagen I (SMD= −2.51, 95%CI: −3.93, −1.09; *p* = 0.0005). Collagen III levels were analyzed in four studies [21,22,24,27], and the results show that the MSC treatment group can lower the level of Collagen III relative to the control group (SMD= −1.50, 95%CI: −2.05, −0.96; *p* < 0.00001). Then, we made the subgroup analysis according to the injection methods, and the result shows that IP injection can reduce the Collagen III level in the MSC treatment group compared to the control group (SMD= −6.96, 95%CI: −11.28, −2.65; *p* = 0.002), but when IV injection was used, there was no discernible difference between the two groups (SMD= −2.40, 95%CI: −6.38, 1.59; *p* = 0.24; Table 3 and Figure 6).

### 3.4. Sensitivity analysis

**Figure 8.**
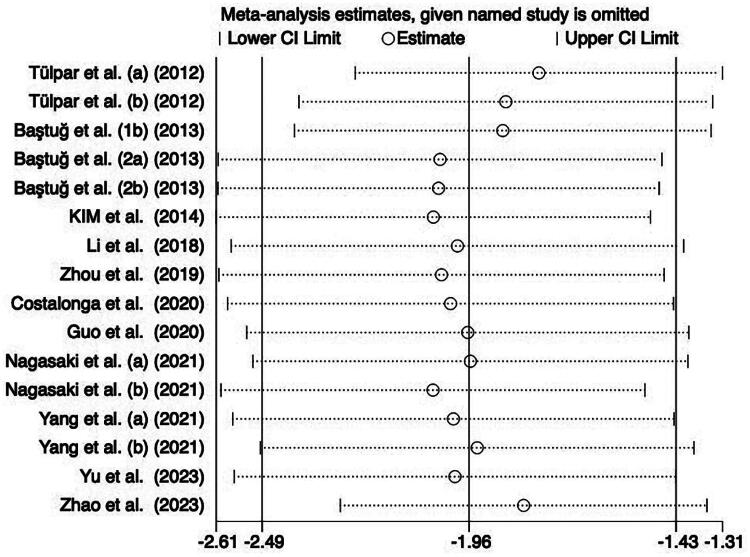
The sensitivity analysis of submesothelial thickness.

We conducted the sensitivity analysis in the D/D0 glucose, α-SMA, and submesothelial thickness. We found that excluding one piece of literature did not markedly change the results of this study, indicating that the results showed good stability in the included studies (Figure 8).

### 3.5. Meta-regression

We conducted a Meta-regression on α-SMA, D/D0 of glucose, and submesothelial thickness metrics to investigate the factors contributing to the high heterogeneity. The results indicated that the source of MSC, the injection method, the injection dose, and the experiment animals were not responsible for the high heterogeneity. So, the high heterogeneity might be caused by the other compounding factors in the literature, such as different MSC culture conditions, different methods of inducing animal models, and different MSC production techniques.

### 3.6. Publication bias

**Figure 9.**
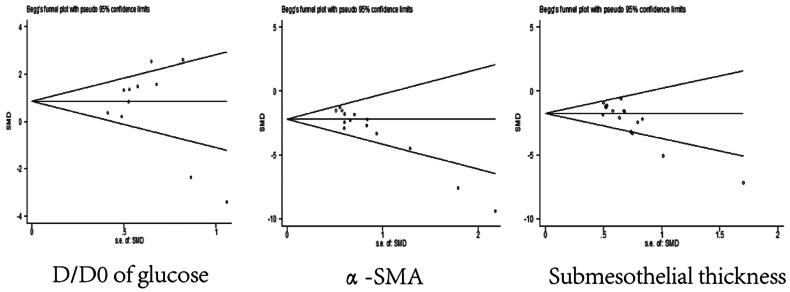
Publication bias.

We assessed publication bias in D/D0 of glucose, and the results show that studies are symmetrically distributed in the funnel plot, and the Begg test(*p* = 0.755)and Egger test(*p* = 0.434)indicate that the studies don’t have the publication bias. After that, the publication bias was also performed in α-SMA and submesothelial thickness (SMC), and the results show that there was publication bias among those indicators (α-SMA: Begg test: *p* = 0.001, Egger test: *p* < 0.0001; SMC: Begg test: *p* = 0.002, Egger test: *p* < 0.0001; Figure 9).

## 4. Discussion

PF is a serious complication of long-term PD. PF can be caused by various factors, including biocompatible dialysate components, uremic toxins, refractory or recurring infectious peritonitis, and chronic inflammation [4,34,35]. The pathophysiologic features of PF including the loss of peritoneal mesothelial cells, an abnormal increase in α-SMA, increased collagen accumulation, and thickening of the submesothelial dense layer [4,5]. MSCs are pluripotent adult stem cells that primarily come from umbilical cord blood, bone marrow, adipose tissue, and placenta [7–9]. MSCs have regenerative, immunomodulatory, and antifibrotic capabilities. A lot of studies have demonstrated that MSCs mediate their therapeutic effects by releasing a series of cytokines and growth factors through the paracrine pathway [36–38]. Stem cell treatment has been shown to be effective in the treatment of multi-system fibrosis of the blood, heart, liver, and kidneys [10–12,39]. Existing studies have suggested that MSCs can alleviate PF, including the following mechanisms: Ueno et al. and KIM et al. [16,18] believe that MSCs secrete hepatocyte growth factors through the paracrine pathway to reduce peritoneal injury. Jiao et al. [40] and Huang et al. [41] suggested that MSCs secrete exosomes to attenuate EMT and reduce PF *via* the AKT/FOXO signaling pathway and the Wnt/β-catenin signaling pathway. Yang et al. [25] found that MSCs can secrete IL-6 to upregulate Arg-1 gene expression in the macrophages to promote M2 macrophage polarization to ameliorate PF. Thus, we have summarized the above studies and made a meta-analysis to evaluate the efficacy of the MSCs in treating PF animal models, which could be an important guide for future clinical trials.

The effectiveness of MSC in treating PF in animal models was examined in this meta-analysis using data from 15 publications. Different sources of MSCs are included in this meta-analysis, such as Rat-BM-MSCs, Rat-ADSCs, hUMSCs, human-pMSCs, and Rat-UCMSC. We also included two MSC exosome trials to treat PF. In addition, this meta-analysis included different injection modalities, MSC doses, the source of MSC, and experimental animals. Due to the large number of compounding factors, we conducted a subgroup analysis of these factors to explore their role and sources of heterogeneity. Firstly, we performed subgroup analyses of injection modality. The results indicate that IV injection could reduce the level of IL-6 and TNF-α, while IP injection is not notable in IL-6 and TNF-ɑ among the MSC therapy group and control group. IP injection could improve the level of D/D0 of glucose and reduce the level of Collagen III, while IV injection has no statistical significance in D/D0 of glucose and Collagen III. IP and IV injection decreased TGF-β, D/P of Cr, fibronectin, submesothelial thickness, α-SMA levels, and increased UF levels. Different injection methods lead to different outcomes in individual indicators, which can be attributed to the small number of included literature and incomplete data collection. Therefore, a large number of basic studies are needed in the future to explore the most suitable injection method. Secondly, we performed a subgroup analysis of the source of MSC. The result shows that MSC derived from rats and humans can reduce TGF-β, D/P of Cr, submesothelial thickness, and α-SMA, which means whether MSCs derived from rats or humans can exert its therapeutic function in PF rat/mice models. Thirdly, we performed a subgroup analysis of experimental animals, and we found that MSCs can increase the UF and D/D0 of glucose as well as reduce submesothelial thickness and α-SMA in the rats and mice models, which means MSCs can exert their therapeutic effect whether rats or mice are used as experimental animals. Lastly, we conducted a subgroup analysis of MSC dosages. The outcomes indicate that there is no statistically significant difference in submesothelial thickness in dose 2. However, using dose 1, dose 3, and dose 4 could reduce D/D0 of glucose, α-SMA, and submesothelial thickness. In future research, exploring the optimal dosage for MSCs is important. Determining the smallest effective dose could reduce the financial burden on patients and effectively treat the disease.

The majority of the included literature in this meta-analysis has high heterogeneity, and some of the metrics have publication bias, which can be attributed to the following reasons: (1) some of the included literature only reported positive outcomes and not negative outcomes; (2) we only included English language literature and excluded other languages such as Chinese; (3) few randomized controlled animal experiments were included; (4) we excluded literature that was relevant to the topic but could not extract data; (5) different ways to induce PF in animal models, different infusion methods of MSCs and different culture conditions in the literature may lead to higher heterogeneity and different results.

Because the number of clinical trials is restricted, the data included in this meta-analysis are from animal researches. However, there are still certain challenges when converting the outcomes of animal investigations into clinical studies, such as: (1) PD patients may be associated with other underlying diseases, and whether these diseases may influence the effect of MSCs needs further investigation; (2) the most suitable dose, the most suitable route of administration and the time window of treatment for the optimal effect of MSCs need to be further studied; (3) *in vitro* expansion technology, transport and storage conditions, extraction and preparation methods of MSCs are still unstandardized. (4) Then, donor-to-donor variability in MSCs may impact therapeutic efficacy. Donor heterogeneity is a significant factor in determining the therapeutic efficacy of MSCs, which in turn affects their functional properties and clinical results. This variability originates from both inter-donor variations and intra-donor discrepancies, which significantly affect essential facets of MSC behavior. Inter-donor variability encompasses two primary components: functional discrepancies and gene expression profiles. Research indicates that MSCs derived from distinct donors demonstrate divergent immunomodulatory capabilities and proliferation rates, which may result in inconsistent clinical outcomes [42]. Transcriptomic analyses have demonstrated that MSCs from different donors exhibit differential gene expression, thereby influencing their responsiveness to inflammatory stimuli and overall therapeutic potential [43]. Furthermore, intra-donor variability include two parts: collection timing and environmental sensitivity. Variability may also arise among MSCs of a single donor, as evidenced by variations in cell viability and functional assessments conducted on samples collected at different time points [44]. Different donor-derived MSCs respond differently to hypoxic environmental conditions, influencing the potential for angiogenesis and therapeutic efficacy in limb ischemia [45]. Thus, a great deal of basic and clinical studies are needed to perform to further enhance the life quality of PD patients.

Meta-analyses of animal studies play a crucial role in ­clinical research by providing more precise estimates of intervention effects, improving methodological rigor, ensuring adequate statistical power, and enhancing the reliability of data. These attributes collectively facilitate the effective translation of findings to human clinical trials. In this meta-analysis, we evaluated inflammatory cytokines and growth factors (such as IL-6, IL-8, TNF-α, VEGF, and TGF-β), peritoneal function, mesenchymal markers, and fibrotic indicators, demonstrating that MSCs can alleviate PF in animal models. The metrics summarized in this study may serve as valuable reference points for future research endeavors. For instance, researchers could consider enhancing the anti-inflammatory and anti-fibrotic properties of MSCs through biotechnological modifications to further alleviate PF in PD patients. Additionally, exploring the combined use of MSCs with specific drugs that improve peritoneal function indicators could lead to more effective therapeutic outcomes. Furthermore, the assessment on fibrosis and mesenchymal markers presented here provide important insights into the pathophysiological mechanisms of MSC therapy, offering a foundation for future studies. Ultimately, these advancements may bring renewed hope to PD patients.

## 5. Conclusions

This meta-analysis shows that MSC treatment could decrease the level of IL-6, IL-1β, TGF-β, TNF-α, D/P of Cr, Fibronectin, submesothelial thickness, α-SMA, Snail, Collagen I, and Collagen III. It can also increase the levels of UF, D/D0 of glucose, and E-cadherin. This study provides information for further exploration of MSC therapy for PF in future studies. However, due to limitations in the amount and quality of included articles, the current study’s conclusions will need to be validated by a significant number of clinical trials.
